# Production of Glucose from PEG-Based Pretreated Green Coconut Fiber and Synthesis of Lignin Nanoparticles for Stabilization of O/W Pickering Emulsion

**DOI:** 10.1021/acsomega.5c07310

**Published:** 2025-11-14

**Authors:** Cleitiane da Costa Nogueira, Amanda Gabriela Viana Fabrício, Gabriela Guimarães Lourenço, Íthalo Barbosa Silva de Abreu, Thelma Sley Pacheco Cellet, Emmanuel Damilano Dutra, Jackson Araújo de Oliveira, Domingos Fabiano de Santana Souza, Carlos Eduardo de Araújo Padilha

**Affiliations:** 1 Laboratory of Alternative Energy and Transport Phenomena, Chemical Engineering Department, Federal University of Rio Grande do Norte (UFRN), Natal, RN 59078-970, Brazil; 2 Research Group On Biomass Energy, Department of Nuclear Energy, Federal University of Pernambuco (UFPE), Recife, PE 50670-901, Brazil; 3 Polymeric and Composite Materials Group, Department of Chemistry, State University of Maringá (UEM), Maringá, PR 87020-900, Brazil

## Abstract

Solvolysis processes or organosolv pretreatments alone have been widely used as a way to obtain glycolated lignins, a type of lignin with thermoplastic properties. Despite this interest, there are still many gaps regarding the byproducts generated and other potential destinations for glycolated lignins, which must be addressed for the process to reach a commercial level. In the present study, the authors investigated the enzymatic digestion of green coconut fiber pretreated with polyethylene glycol (PEG) 400 and sulfuric acid, as well as the performance of glycolated lignin as a stabilizer of o/w Pickering emulsions, a notable application for lignins. The PEG-based organosolv pretreatment promoted extensive delignification (reduction of Klason lignin from 35.7 to 29.7%) and enabled greater cellulose enrichment than pretreatment with sulfuric acid alone (33.4 vs 44.8%). In terms of enzymatic digestibility, glucose release from the organosolv-pretreated sample was 4.3- and 2.8-fold higher than that from the untreated and acid-pretreated samples, respectively. Glycolation in green coconut fiber lignin was confirmed by FTIR and thermogravimetry analyses. Glycolated lignin nanoparticles were successfully prepared and exhibited a rounded shape and size in the range of 30–50 nm. Regardless of the o/w system (including toluene/water, soybean oil/water, and *n*-octanol/water), glycolated lignin nanoparticles led to higher emulsification index (up to ∼80% after 14 days) and better stability than lignin nanoparticles isolated from acid pretreatment. This study confirms the versatility of PEG pretreatment and demonstrates that it can serve as the basis for an alternative biorefinery for lignocellulosic biomass.

## Graphical abstract

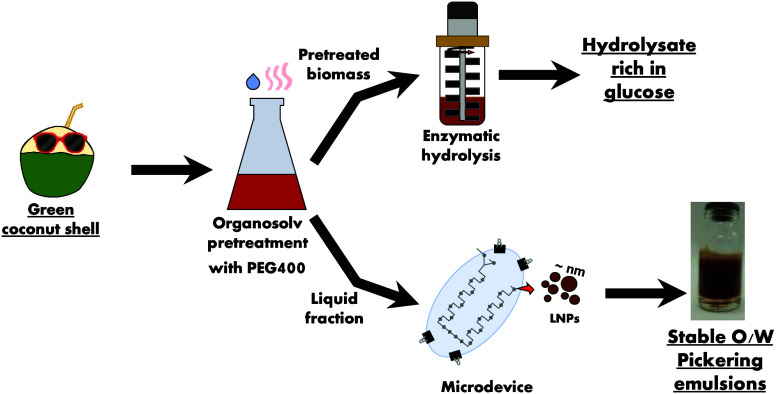

## 1 Introduction

Cellulosic ethanol, also known as second-generation ethanol, is produced from agro-industrial waste, primarily lignocellulosic waste. The use of this resource avoids competition with food production and can help with energy security in times of high carbon emissions penalties.^1^ Tropical countries, such as Brazil, have lignocellulosic waste with potential applications for cellulosic ethanol, including green coconut fiber.^2,3^ Green coconut is responsible for supplying coconut water and coconut pulp derivatives, which are widely consumed by people around the world.^4^ However, after processing green coconuts, their shells are considered waste and comprise 80% of the initial mass of the fruit. According to the NationMaster Web site, the annual generation of coconut residues is estimated at 50 megatons,^5^ which represents a significant burden on the region’s solid waste treatment systems and an opportunity to obtain cellulosic ethanol.

However, the conversion of green coconut fiber into ethanol requires the application of pretreatments to break the recalcitrance of the biomass. Chemical pretreatments involve incubating lignocellulosic residues with catalysts and/or organic solvents, a process widely used to solubilize lignocellulose components and enhance cellulose accessibility.^6^ Biomasses obtained after chemical pretreatment generally have digestibility several times higher than native or ground biomass. Among the various chemical pretreatments, organosolv pretreatment stands out for its effectiveness in disorganizing biomass and facilitating fractionation.^7^ The organic solvents in this pretreatment play a crucial role in facilitating the action of catalysts on the lignin-carbohydrate complex, while also helping to solubilize lignin fragments and prevent redeposition onto biomass.^8^ In the presence of acid catalysts, organosolv pretreatments, in addition to delignification, are capable of removing hemicellulose and, therefore, enriching the pretreated biomass in cellulose.^9,10^ Successful cases of organosolv pretreatments using green coconut waste have been previously published, with an emphasis on systems based on glycerol and ethanol, as reported by Ebrahimi et al.,^2^ Padilha et al.,^3^ and Abd Latif et al.^11^ Indeed, the low cost of glycerol justifies its dominance in the literature, but it would be interesting to explore the performance of other organic solvents. In this context, it is noteworthy to highlight the role of polyethylene glycol (PEG), a polymer composed of ethylene glycol units. PEG has been widely used to enhance the enzymatic hydrolysis of biomass by mitigating the nonproductive adsorption of cellulases on lignin, particularly in pretreatments. Kim et al.^12^ and Qing et al.^13^ suggest the addition of small doses of PEG to increase the solubility of lignin and pseudolignin in hydrothermal and acid pretreatments, while Lai et al.^14^ investigated the modification of lignin in the biomass structure using PEG epoxide. Despite these efforts and the recognized ability to solvate lignin, there are still few studies using PEG as an organic solvent for organosolv pretreatments and examining its performance in the delignification of biomass, especially green coconut fiber. Nor have results been presented on the enzymatic digestibility of organosolv pretreated biomass based on PEG, and whether this route is comparable to the conventional addition of the chemical only in enzymatic hydrolysis.

Increasingly, studies on cellulosic ethanol have demonstrated the value of other process streams, particularly lignin, following organosolv pretreatments. Compared to other technical lignins, organosolv lignins commonly present greater biocompatibility due to the absence of sulfur, greater solubility in organic solvents, and greater antioxidant activity.^15,16^ In addition to providing solubilization, some organic solvents during organosolv pretreatment behave as nucleophiles, acting to stabilize β-O-4 bonds in lignin and providing a structure with a lower degree of condensation.^17^ If, on the one hand, this mechanism of organosolv pretreatment is attributed to the purity of lignin, on the other hand, it may be a means of adding solvent properties to lignin. Recently, authors such as Nge et al.,^18^ Ju et al.,^19^ and Yamada et al.^20^ have proposed the production of glycolated lignins through PEG-based solvolysis. This organosolv pretreatment utilizes only low-molecular-weight PEGs as the liquid bulk and strong acids as the catalyst. Glycolated lignins are characterized by their thermoplastic nature, which is attributed to the presence of PEG chains within the lignin structure, allowing them to be used as a lubricant or ingredient in plastic components for a wide range of purposes.^18,21^ Although these applications are exciting, the functionalization of lignin with PEG can unlock benefits in the stabilization of Pickering emulsions, a prominent application for lignins. Pickering emulsions consist of emulsions stabilized by nanoparticles, which migrate to the interface, thereby limiting the coalescence of droplets in the dispersed phase.^22^ In particular, the grafting of PEG chains onto lignin can generate nanoparticles with a more hydrophilic surface, which can tailor their wettability and consequently alter the stability, as well as the properties, of Pickering emulsions. Studies have already addressed the improvement of lignin amphiphilicity in the presence of PEG, as seen in Wen and Fu;^23^ however, no consistent investigation has been carried out so far with lignins recovered directly from a PEG-based organosolv pretreatment.

Thus, the present work investigated an integrated scheme to obtain glucose and glycolated lignin from green coconut shells with PEG-based organosolv pretreatment as the key step. The pretreatment consisted of an acidification step using PEG 400, followed by washing with sodium hydroxide. Enzymatic hydrolysis tests were conducted under different solid loadings, and comparisons were made with other types of pretreatment. The lignin isolated from the organosolv pretreatment of GCF was compared with acid lignin from the same biomass. Then, organosolv lignin nanoparticles were prepared through solvent displacement and used for the stabilization of o/w emulsions.

## 2 Materials and Methods

### 2.1 Chemicals

Citric acid, sodium citrate, sodium chloride, d-glucose, ethanol, sodium azide, sodium hydroxide, polyethylene glycol 400 (PEG 400), sulfuric acid, toluene, and n-octanol were purchased from Synth (São Paulo, Brazil). 3,5-Dinitrosalicylic acid, acetonitrile, and a glucose oxidase-peroxidase (GOD-POD) enzyme kit were purchased from Sigma-Aldrich (MO, USA). Soybean oil was purchased from the local market.

### 2.2 Biomass and Enzymes

Green coconut husks were collected from urban areas of Natal, a city in northeastern Brazil. The material was cut with a machete, and the pieces were washed with running water and then dried at 50 °C for 72 h in a TEM-394/I circulating air oven (TECNAL, Brazil). The green coconut pieces will be ground in a TE-680 knife mill (TECNAL, Brazil) to ∼ 0.85 mm (20 mesh) and stored in plastic bags. The enzyme cocktail Cellic Ctec2 (∼120 filter paper units/mL; ∼ 120 FPU/mL) was used in the enzymatic hydrolysis experiments of cellulose-rich materials.

### 2.3 Organosolv Pretreatment of Green Coconut Fiber

The organosolv pretreatment of green coconut fiber was used as a medium to generate cellulose-rich solid fraction and PEGylated lignin samples as described in Figure 1. Initially, 10 g of GCF (on a dry basis), 100 g of PEG 400, and 0.3 g of sulfuric acid were mixed in a 500 mL Erlenmeyer flask. The flask was then autoclaved at 130 °C for 120 min. The resulting slurry was mixed with 125 mL of 0.4 M NaOH solution for 5 min at room temperature (∼25 °C), and then the solid and liquid fractions were separated via cloth filtration. To prepare the solid fraction for enzymatic hydrolysis, the material was washed six times with 100 mL of tap water in each round and subsequently dried at 60 °C in an air circulation oven. Acid pretreatment was prepared to perform an enzymatic digestibility comparison, and it was performed by replacing PEG 400 with water. An organosolv pretreatment without sulfuric acid was also performed.

**Figure 1:**
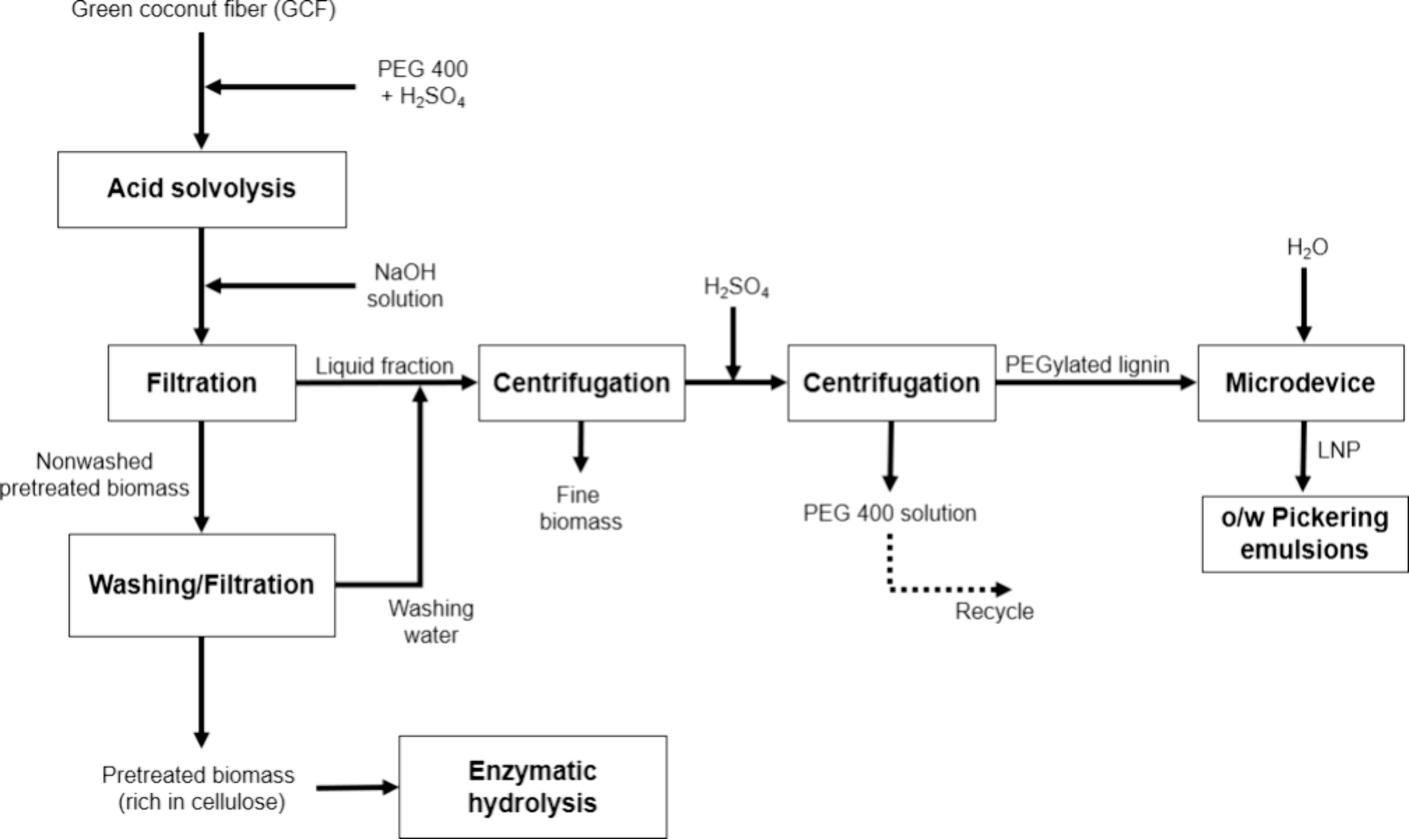
Representation of the steps performed for the organosolv pretreatment of green coconut fiber and recovery of PEGylated lignin.

To increase lignin yield, the first wash water was added, along with the liquid fraction of the pretreatments, as a lignin source. The liquid was previously centrifuged at 1,500 × g in 50 mL conical tubes using a SL-700 centrifuge (SOLAB, Brazil) to remove particulates that had passed through the cloth opening. Then, 5 M sulfuric acid was added to the liquid to adjust the pH to 2.0, and the mixture was left under magnetic stirring for 30 min. The glycolated lignin was separated from the liquid phase using a centrifuge at 1,500 × g, washed three times with acidified water (pH 2.0), and dried in an air circulation oven at 60 °C for 24 h. The lignin recovered from the pretreatment in the absence of PEG 400 was called acid lignin and it followed the same steps as organosolv lignin. The description of lignin nanoparticle preparation has been added to Section 2.5.

### 2.4 Enzyme Hydrolysis of Organosolv Pretreated GCF

The production of glucose from pretreated GCF was performed using enzymatic hydrolysis. In a 25 mL Erlenmeyer flask, pretreated GCF (with a working volume of 5 mL), citrate buffer (50 mM, pH 5.0), and sodium azide (0.01%, w/v) were added to the reaction medium, along with 20 FPU/g of Cellic Ctec 2 cellulases. The flasks were incubated on a rotary shaker at 50 °C and 150 rpm for 48 h. Samples were collected at 0 and 48 h, and the enzymes were promptly inactivated by heating prior to glucose quantification in the supernatant. The experiments were performed under solid loadings of 10% and 20% (w/v) of pretreated GCF. To compare the increase in digestibility resulting from pretreatment with PEG 400, experiments were also conducted using untreated, acid-free organosolv pretreated, and acid-pretreated GCF (obtained in Section 2.3) as substrates. Particularly, in the experiments with untreated and acid-pretreated GCF, Tween 80 and PEG 400 were added to the reaction medium at a dosage of 0.1 g per g of substrate.

### 2.5 Synthesis of Lignin Nanoparticles in a Microdevice

The preparation of LNP began with the solubilization of 1 g of PEGylated lignin in a 100 g PEG:water mixture (8:2 w/w). The mixture was stirred in a beaker under magnetic stirring for 24 h. Then the residual solids were removed by filtration using filter paper, followed by filtration with a 0.45 μm membrane. A homemade microdevice was designed to establish contact between the lignin solution and water, facilitating the preparation of LNP. The apparatus consisted of a Parafilm M layer (∼127 μm) cut from a Scanncut SDX85 machine, 10.7 × 5.7 cm oval-shaped acrylic plates, and clips. The cut path consisted of a Y-junction and 1 mm-thick zigzag channels interspersed with 3 mm-thick openings to facilitate mixing between the phases. A syringe pump and a peristaltic pump were used to feed the lignin solution and acidified water (pH 2), respectively. The general apparatus and the cross-sectional path are shown in Figure 2. For LNP preparation, the volumetric flow rate of the lignin solution was set at 400 μL/min, while the flow rate of acidified water (pH 2.0) was set at 4 mL/min. The generated LNPs were collected in a beaker and then centrifuged and washed with acidified water to remove contaminants. The LNPs were resuspended in acidified water to simplify their use in the Pickering emulsion, and the LNP content was evaluated after drying at 105 °C.

**Figure 2:**
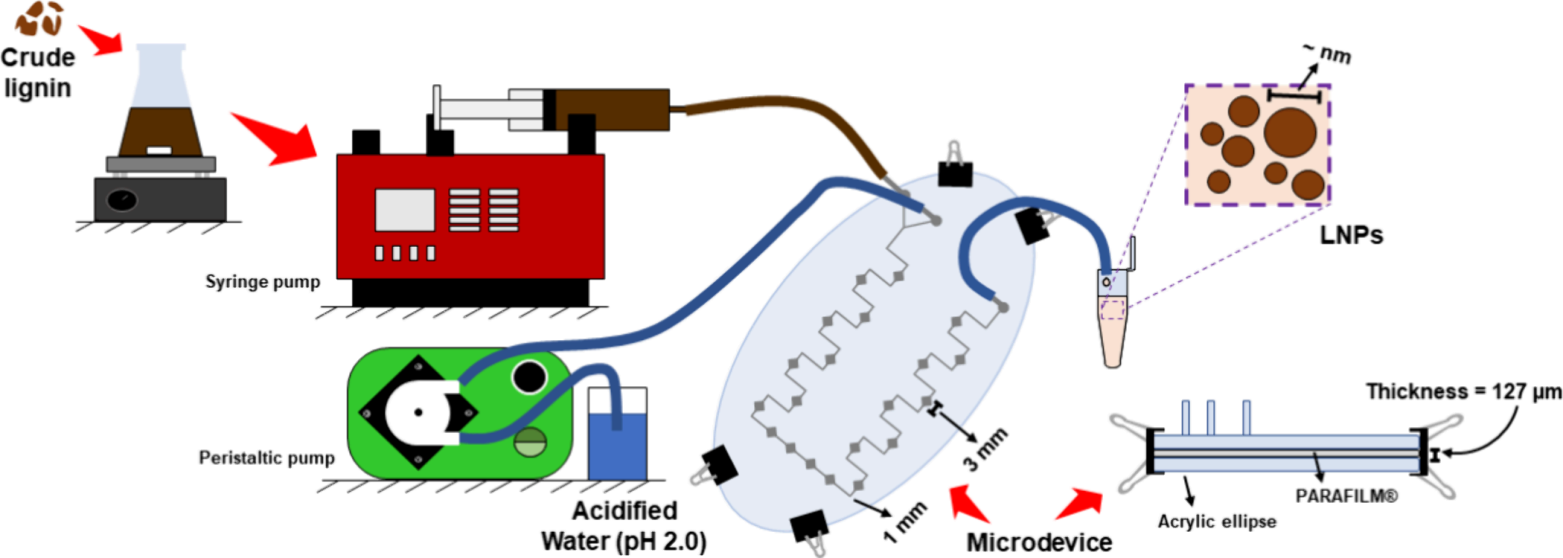
Representation of the microdevice used in the preparation of LNPs from green coconut fiber.

### 2.6 Preparation of O/W Pickering Pickering Emulsions

The Pickering emulsion experiments were performed (in triplicate) using different hydrophobic solvents (toluene, soybean oil, and n-octanol) with green coconut LNP as the stabilizer. The LNP suspensions were diluted with acidified water in test tubes to reach a volume of 1 mL under concentrations of 0.5, 1.0, 2.0, and 5.0 g/L. Then, 1 mL of the hydrophobic solvents was slowly added, and the tubes were homogenized by vortexing for 30 s. After 1 and 14 days at room temperature, emulsification indices were evaluated using a caliper (eq 1), and droplet sizes were assessed using a K55-TA optical microscope (Kasvi, Brazil). To evaluate the effects of ionic strength on the performance of glycolated lignin, experiments were conducted in the n-octanol/water system at ionic strengths of 25, 50, 100, and 250 mM sodium chloride. Droplet size results were presented as a cumulative size distribution, as calculated in eq 2.

$$EI(\%)=\frac{emulsified\;layer\;(mL)}{total\;volume\;(mL)}\times 100$$ 1

$$q_{i}(\%)=\left(\frac{1}{total\;number\;of\;droplets}\times 100\right)+q_{i-1}(\%)$$ 2

### 2.7 Analytical Methods

#### 2.7.1 Characterization of Untreated and Pretreated Coconut Fiber

The chemical compositions of untreated and pretreated GCF were determined in terms of cellulose, hemicellulose, Klason lignin, ash, and extractives. The methodologies used were adapted from Sluiter et al.^24–26^ Crystallinity was analyzed using an XRD-6000 X-ray diffractometer (Shimadzu, Japan), which was operated at a voltage of 40 kV and a current of 30 mA, utilizing Cu Kα radiation. The following equation calculates the crystallinity index (CrI, %):

$$CrI\left(\%\right)=\left(\frac{I_{002}-I_{am}}{I_{002}}\right)\times 100$$ 3

Where I002 is the intensity value corresponding to plane 002 (2θ = ∼ 22.6°), and Iam is the intensity value of the valley between the peaks of planes 002 and 001 (2θ = ∼ 18.7°).

#### 2.7.2 TGA

The confirmation of lignin PEGylation was evaluated using thermal sensitivity analysis on a DTG-60 equipment (Shimadzu, Japan). The equipment was operated within a temperature range of 25 to 700 °C, with a sample mass of 10 mg, a heating rate of 10 °C/min, and in an inert environment (nitrogen gas).

#### 2.7.3 FTIR and Zeta Potential

The FTIR profiles of the lignin samples (organosolv lignin and acid lignin) were evaluated on a FTLA 2000 spectrometer (ABB Bomem Inc., Canada) using the range of 4000 to 400 cm^–1^ with a resolution of 4 cm^–1^. The values of zeta potential were determined at pH 2.0 using 90Plus/Bi-MAS Zeta Plus (Brookhaven, USA) after ultrasonication. The pH adjustment of the lignin suspensions was performed using diluted solutions of sulfuric acid and sodium hydroxide.

#### 2.7.4 Transmission Electron Microscopy

The size of the LNPs was evaluated by transmission electron microscopy (TEM) analysis using a JEM 1400 microscope (JEOL, Japan) at an acceleration voltage of 120 kV.

### 2.8 Statistical Analysis

To evaluate the significance of effects in the enzymatic hydrolysis and emulsification index experiments, Tukey tests were performed using Statistica 7.0 software (StatSoft/USA), at a 95% confidence level (*p* < 0.05).

## 3 Results and Discussion

### 3.1 Chemical Composition and Crystallinity Index of GCF Samples

Organosolv pretreatment was effective in modifying the chemical composition of GCF, as shown in Table 1. GCF is a biomass recognized for its high extractives content (in this case, 16.5%), as it comprises the powder of the mesocarp and epicarp of the fruit. Lima et al.^27^ reported the presence of phenols, tannins, leucoanthocyanidins, flavonoids, triterpenes, steroids, and alkaloids in the mesocarp. After organosolv pretreatment, due to the intense disruption of the lignocellulosic matrix, the extractives content decreased to only 4.1%. Ash showed a slight decline after organosolv pretreatment of GCF, reducing from 3.2% to 2.5%. The acidic environment favors the leaching of inorganic materials in the biomass, especially metal oxides, leading to conversion into water-soluble salts and accumulation in the liquid fraction of the pretreatment.^28^ As a reference, pretreatment with acid alone was successful, and the extractives and ash contents were reduced to 3.1% and 2.6%, respectively. Acid pretreatment also led to a decline in hemicellulose content, from 15.8% to 10.2%. In contrast, the organosolv pretreated GCF showed practically no changes due to the absence of water in the liquid phase of the pretreatment. The same occurred with the sample obtained from acid-free organosolv pretreatment. It is noteworthy that the alkaline washing step does not provide a drastic enough condition to alter the hemicellulose content, as indicated by studies on xylan isolation.^29^

**Table 1:** Chemical Composition and Crystallinity of Untreated and Pretreated Green Coconut Fiber

sample	yield (%)	cellulose (%)	hemicellulose (%)	Klason lignin (%)	extractives (%)	ashes (%)	CrI^a^ (%)
untreated GCF		28.8 ± 1.1	15.8 ± 1.4	35.7 ± 1.3	16.5 ± 0.1	3.2 ± 0.2	31.2
acid pretreated GCF	67.7	33.4 ± 4.4	10.2 ± 1.1	45.6 ± 0.2	3.2 ± 0.5	2.6 ± 0.1	36.1
organosolv pretreated GCF without acid	77.1	32.1 ± 1.5	16.2 ± 0.4	33.8 ± 1.8	8.1 ± 0.8	3.3 ± 0.2	33.5
organosolv pretreated GCF	61.3	44.8 ± 1.6	15.2 ± 2.1	29.7 ± 0.4	4.1 ± 0.8	2.5 ± 0.1	43.4

a CrI – crystallinity index.

The roles of pretreatments differ in terms of their action on lignin. Organosolv pretreatment reduced lignin content from 35.7% to 29.7%, while lignin content increased after acid pretreatment to 45.6%. The use of strong acid as a catalyst in organosolv pretreatment is providential, since it promotes extensive disruption of the biomass and facilitates the release of lignin and fragments to the solvent-rich liquid phase.^2^ This argument explains the fact that the lignin content in the acid-free organosolv pretreated sample was 33.8%, still close to that of the untreated sample. Hernández-Ramos et al.^30^ have already reported that PEG 400 is a polyether with excellent lignin solubilization capacity. In acid pretreatment, on the other hand, lignin fragments generated by the acid tend to interact with each other, leading to condensed structures that are distributed on the surface of the pretreated biomass.^17^ Other papers have reported lower Klason lignin values in green coconut fiber obtained after combined pretreatments.^31,32^ However, the differences in the findings of the present study stand out, as only one alkaline washing was performed. Possibly, the fact that heating was not required prevented sodium hydroxide from promoting extensive lignin depolymerization and its detachment from the lignocellulosic matrix. Based on Nge et al.,^18^ the intention of washing with an alkaline agent after organosolv pretreatment was only to detach the lignin from the biomass surface and displace it to the liquid bulk without compromising the PEGylation itself.

As a consequence of removing amorphous components, both pretreatments promoted increases in cellulose content. The cellulose content increased from 28.8% to 33.4% in the acid pretreatment and 44.8% in the organosolv pretreatment. As in hemicellulose, the absence of water in the reaction environment limits the depolymerization of cellulose and, therefore, the highest retention of the polysaccharide was recorded in the organosolv pretreatment (∼95%). The crystallinity analyses of the green coconut samples corroborated the enrichment of cellulose in the pretreatments. The organosolv pretreated GCF showed a 1.4-fold increase in crystallinity (43.4%) compared to the untreated GCF (31.2%). As a consequence of the lower loss of amorphous components, the acid pretreated sample showed a slightly higher CrI value (36.1%). The acid-free organosolv pretreated GCF showed a CrI of only 33.5%. Studies with effective pretreatments using green coconut fiber have shown similar results, with CrI values of around 40%–50%.^3,32^

### 3.2 Performance Comparison in Enzymatic Hydrolysis

Figure 3 presents the glucose release performance in the enzymatic hydrolysis of different GCF samples. In agreement with the chemical characterization data, the organosolv-pretreated GCF showed better results than the untreated and acid-pretreated samples, regardless of the presence of surfactant. At 10% solids, the hydrolysis of organosolv-pretreated GCF obtained 14.03 g/L, which represents an increase of 4.3 times and 2.8 times compared with the untreated and acid-pretreated samples, respectively. The tests with 20% solids yielded even higher glucose release (26.61 g/L) due to the greater availability of cellulose in the environment.

**Figure 3:**
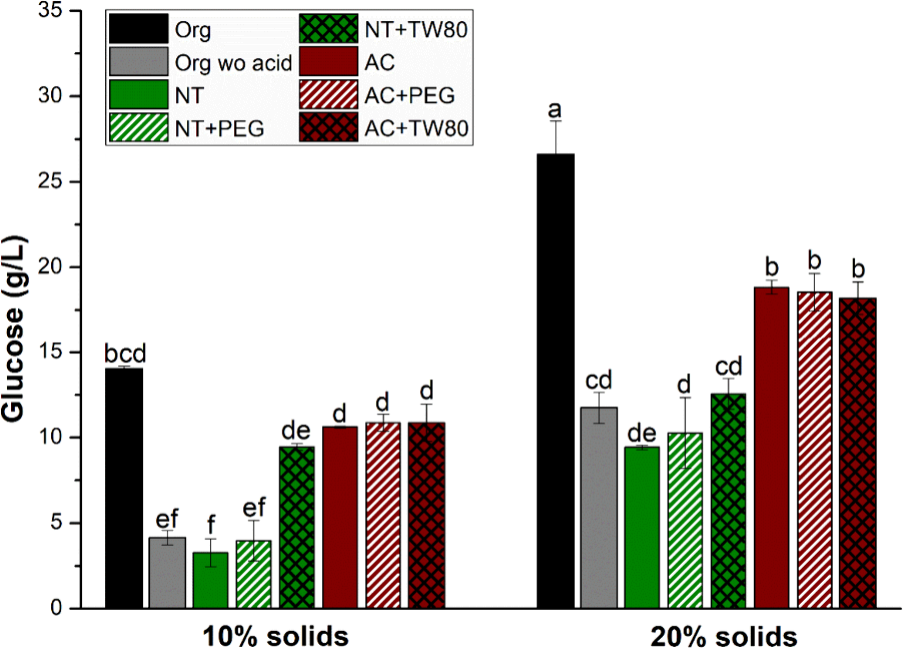
Glucose release during the enzymatic hydrolysis of untreated and pretreated green coconut fiber (GCF) samples. The abbreviations Org, NT, and AC correspond to organosolv pretreatment, untreated, and acid pretreatment, respectively. The abbreviations PEG and TW correspond to polyethylene glycol 400 and Tween 80 additives, respectively. The tests were performed at a temperature of 50 °C, rotation of 120 rpm, and time of 48 h. Equal letters indicate that there are no statistical differences between the experimental conditions at the 5% significance level.

Surfactants are widely used as chemical additives in the enzymatic hydrolysis step to mitigate nonproductive adsorption of cellulases and enhance results in systems with acidic pretreated samples.^13,31^ However, this behavior was not observed in the present study. The addition of Tween 80 was indeed decisive for the increase in glucose release with untreated GCF (from 3.26 to 9.45 g/L with 10% solids). However, it was also not beneficial for the acidic pretreated sample. The addition of PEG did not improve glucose release regardless of the GCF sample and solid loading, confirming the effectiveness of the combination of acid and PEG in organosolv pretreatment. The absence of acid in the organosolv pretreatment led to poor glucose results for both solid loadings (4.13 and 11.73 g/L). It is noteworthy that GCF is a biomass recognized for its high lignin content, and the presence of PEG in the organosolv pretreatment promotes delignification, unlike acid pretreatment, which primarily acts on hemicellulose. Another hypothesis linked to the organosolv pretreatment is the chemical modification of the residual lignin in the pretreated biomass. As with the lignin released in the liquid fraction, it is possible that PEG 400 was grafted onto the chemical structure of lignin by alkoxylation in the α position or by esterification.^17^ Therefore, the residual lignin would have a more hydrophilic nature and would be less capable of establishing hydrophobic interactions between cellulases and lignin, which is a strong contributor to nonproductive adsorption.^14,33^ In Lai et al.,^14^ the authors performed pretreatments of corn stover with in situ glycolation using PEGDE, observing a reduction in the adsorption of cellobiohydrolases and beta-glycosidases. These results are particularly convenient for the field of preparing glycolated lignins, as they also facilitate the debottlenecking of products from pretreated biomass, which was sometimes overlooked in previous papers.

### 3.3 Characterization of Glycolated Lignin and Preparation of Nanoparticles

The lignin isolated from the organosolv pretreatment is shown in Figure S1. This lignin exhibits a dark brown coloration typical of the macromolecule, but it does not present brittle characteristics; rather, it is deformable, similar to PEG of high molecular weights. The recovery of the glycolated lignin was estimated at 7.4 g per 100 g of initial biomass, corresponding to approximately 20.7% of the lignin content, which is in agreement with other reports in the literature. Suzuki et al.^34^ reported recovery values of 12%–42% for glycolated lignins from organosolv pretreatment of cedar wood meal using PEG 400. In terms of charge density, there were practically no differences between the zeta potential values of organosolv lignin (−7.34 mV) and acid lignin (0.91 mV). In order to confirm the grafting of PEG in the lignin recovered from the organosolv pretreatment, FTIR and thermogravimetric analyses were performed and compared with a lignin isolated from PEG-free pretreatment. Unlike acid lignin, organosolv lignin showed peaks in the regions of 1110 and 1252 cm^–1^ in Figure 4B, both relative to the C–O ether bond.^35,36^ Significant changes were observed in the spectrum of organosolv lignin in the regions corresponding to 946 cm^–1^ (C = C bond), 1070 cm^–1^ (primary alcohol C–O bond), and 1350 cm^–1^ (O–H bond),^36^ indicating the presence of glycolation in its structure. In Figure 4C, it is possible to observe that organosolv lignin is more sensitive to heating than acid lignin. An abrupt decline in the mass of organosolv lignin was observed up to 400 °C, a typical region of PEG degradation. TG analysis was also a tool to prove the glycolation of lignins, as in Akhtar et al.^37^ and Cortés-Triviño et al.^38^ In both studies, degradation in the range of 350–400 °C was indicated as evidence of the presence of PEG chains in the lignin structure. In the present study, the mass loss related to PEG degradation was about 14%, indicating that PEG grafting was indeed extensive and is beneficial for the purpose of developing an emulsion stabilizer. After this temperature range, organosolv lignin showed mild mass loss up to 700 °C, as did acid lignin.

**Figure 4:**
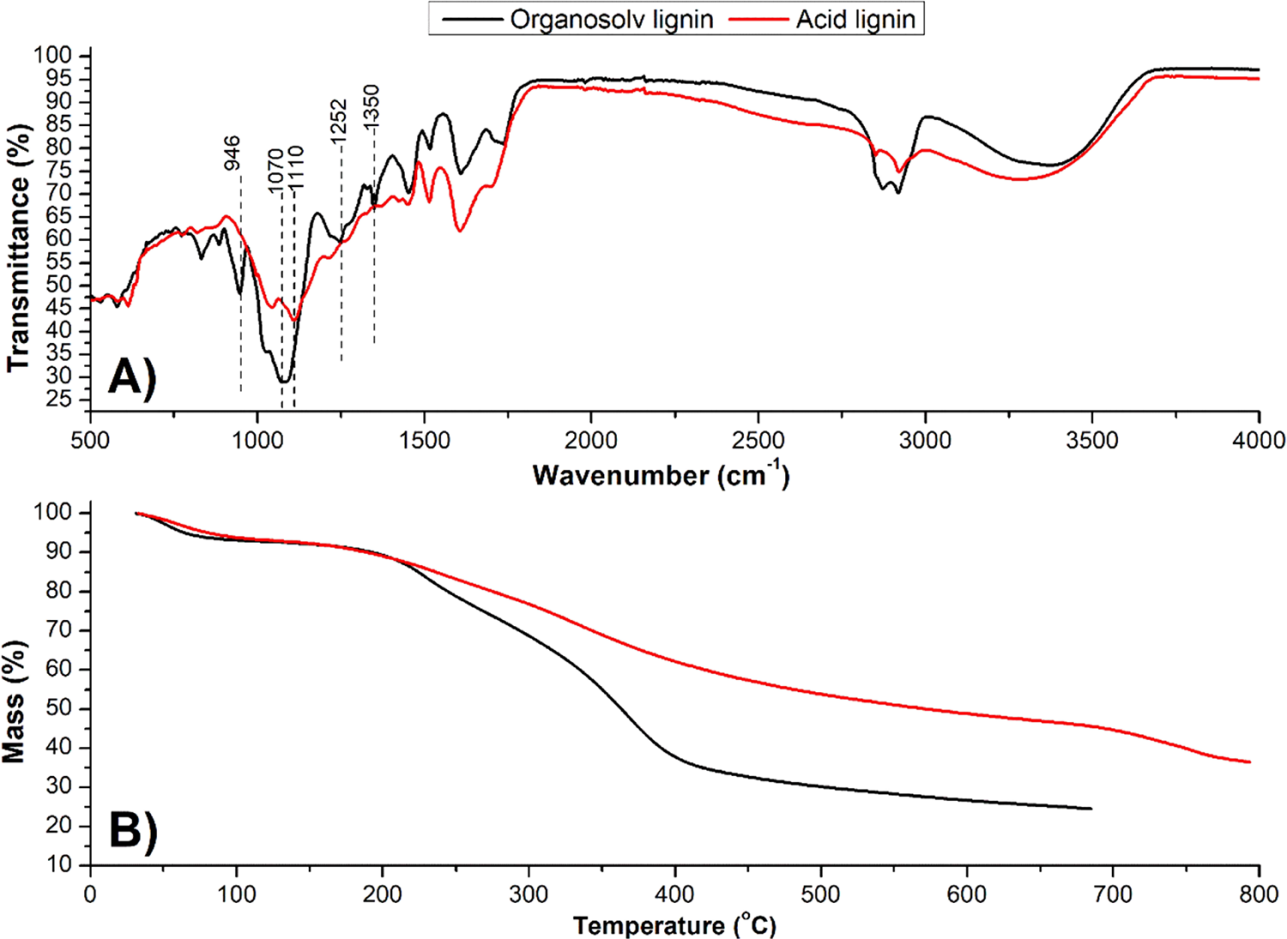
(A) Transmittance profile of organosolv lignin and acid lignin samples from green coconut fiber; (B) Thermogravimetry behavior of organosolv lignin and acid lignin samples from green coconut fiber.

Nanoparticles of organosolv lignin and acid lignin were successfully prepared in the microdevice. Figure 5 shows that the generated nanoparticles had a rounded shape and sizes in the order of 30–50 nm, a notable result in the literature. These dimensions are significantly smaller than those of particles prepared in conventional experiments, such as dripping in static baths and dialysis.^39–41^ Reducing the dimensions of nanoparticles is desirable for the function of a Pickering emulsion stabilizer because it ensures a larger surface area, thereby improving droplet coverage.^42,43^ Due to this need, the role of the microdevice becomes crucial. Microdevices enable high mass transfer rates as well as greater process control, which can lead to good reproducibility in nanoparticle applications.^44^ Future studies can be carried out using the liquid fraction of the organosolv pretreatment directly in solvent displacement, which would reduce the need for drying and lignin resolubilization efforts.

**Figure 5:**
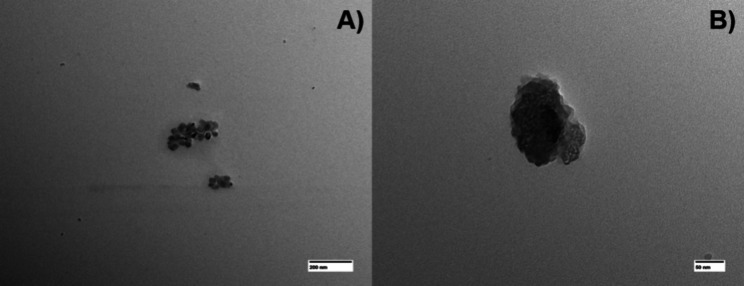
TEM images of organosolv lignin nanoparticle samples (A - 50k×, B – 200k×) prepared in the microdevice.

### 3.4 Performance of Organosolv Lignin Nanoparticles as a Stabilizer of O/W Pickering emulsions

Figure 6 shows the emulsification index values of the different o/w systems in the presence of LNPs. As expected, increasing the LNP dosage improves the stabilization of the o/w systems, regardless of the type of hydrophobic solvent. Nanoparticle concentrations lower than 0.5 g/L were insufficient to cover the available interfacial area and, therefore, presented high coalescence and low stability (data not shown). In turn, from 0.5 g/L onward, it is possible to observe the formation of a film around the droplets, and the increase in nanoparticle concentration changes the thickness of this film, resulting in a larger emulsified layer (up to 80.3%) and smaller droplets with uniform distribution (Figure S2). This network also offers resistance to flocculation and coalescence, thus improving the overall efficiency.^45^ In the n-octanol/water system, the dosage of 1 g/L nanoparticles led to droplets with sizes greater than 200 μm (see Figure S3), while the largest droplets were 50 μm when 5 g/L nanoparticles were used. The same behavior was recorded for the soybean oil/water and toluene/water systems.

**Figure 6:**
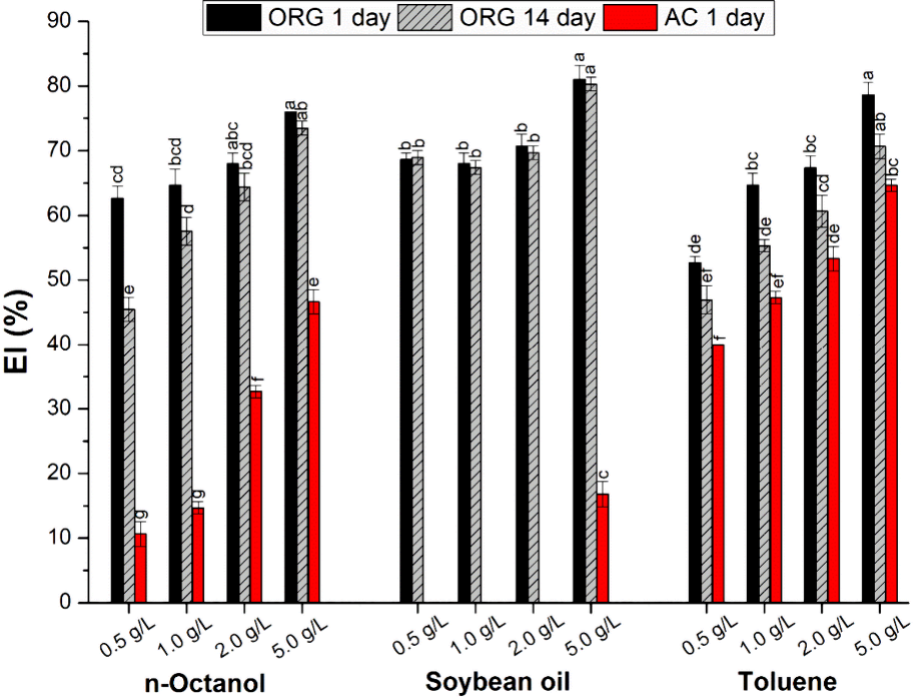
Emulsification index values in the Pickering n-octanol/water (A), soybean oil/water (B), and toluene/water (C) systems under different concentrations of organosolv lignin (ORG) and acid lignin (AC) nanoparticles. All experiments were conducted at a volumetric ratio of 1:1 and room temperature (∼25 °C).

The type of LNP showed a remarkable effect on the stabilization of the Pickering emulsions. In Figure 6, the use of 0.5 g/L organosolv lignin nanoparticles led to an EI of 62.7%, while the acid lignin nanoparticles obtained an EI of only 10% at the same dosage. The acid lignin nanoparticles were ineffective in stabilizing the soybean oil/water system, forming an emulsified layer only at 5 g/L nanoparticles. In turn, the organosolv lignin nanoparticles presented EI higher than 70% and practically no alteration of the system was recorded after 14 days (see Figure 6 and Figure S4). Although the literature reports the low performance of lignins in emulsions with an oil phase of higher polarity, the organosolv lignin nanoparticles also presented a high EI (>53%) in the n-octanol/water system. The wide availability of ether oxygen in PEG provides greater electronegativity and, consequently, amphipathic character to lignin molecules and derived nanoparticles, which is particularly essential in Pickering emulsions.^22^ In Gao et al.,^46^ the authors observed an increase in the EI value and a reduction in droplet size in toluene/water systems due to the increased hydrophilicity of lignins via sulfobutylation. The PEG chains also provide the effect of steric repulsion to the nanoparticles. According to Moreno and Sipponen,^47^ after self-assembly, the grafted hydrophilic polymers accumulate in an outer layer, while the inner portion would be more hydrophobic due to the accumulation of aromatic moieties. This configuration allows LNPs to cover a larger interfacial area under the same stabilizer dosage and, consequently, achieve higher EI values and stability (see Figure 7). This hypothesis also explains the better performance of the organosolv lignin nanoparticles in Pickering emulsions in the presence of sodium chloride. In theory, the addition of sodium chloride negatively affects the adsorption of nanoparticles at the oil–water interface, reducing electrostatic repulsive forces and stimulating hydrophobic interactions that lead to nanoparticle aggregation and flocculation.^22^ The droplet size distribution became increasingly wider as the ionic strength increased in the systems with acid lignin nanoparticles (see Figure S5). However, the droplet size remained practically unchanged with increasing ionic strength in systems containing organosolv lignin nanoparticles. Increased salt tolerance of nanoparticles via PEG grafting was recently reported by Prawatborisut et al.^48^ and Daneshmand et al.^49^

**Figure 7:**
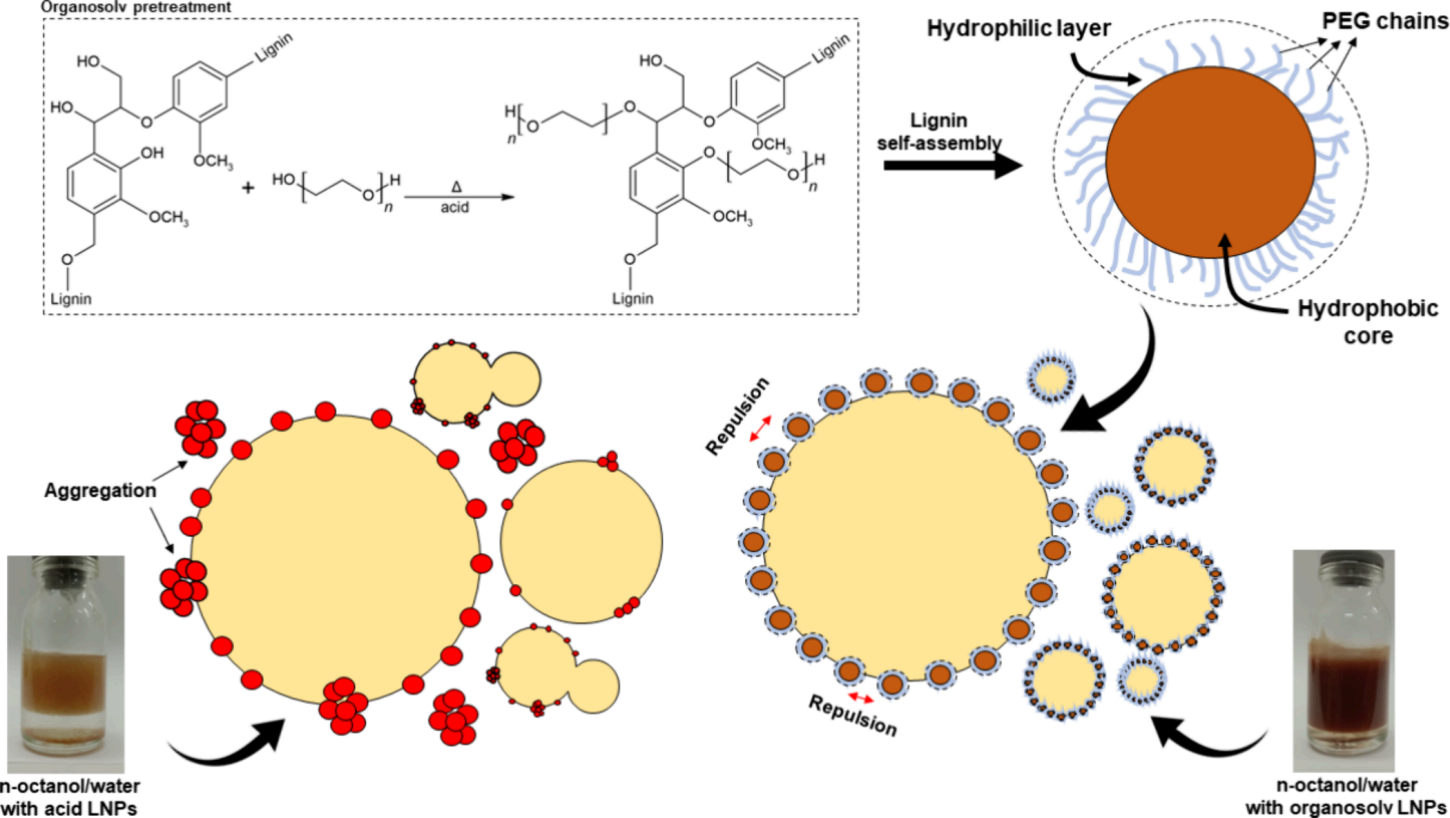
Representation of PEG grafting on the structure of lignins during organosolv pretreatment and stabilization mechanism of LNPs in Pickering emulsions.

These results demonstrate that researchers can overcome the limitations of isolated macromolecule structures and contribute to efforts to valorize lignin in industrial chains. Since lignin exhibits bioactive and anti-UV properties,^10,15^ the success of lignin-stabilized Pickering emulsions could facilitate the development of numerous value-added products with potential applications in the food, cosmetics, and pharmaceutical industries.

## 4 Conclusions

The PEG-based pretreatment promoted cellulose enrichment in the pretreated green coconut fiber due to high delignification. Consequently, the organosolv pretreated green coconut fiber allowed good digestibility even under 20% solids, being several times superior to the results with the untreated and acid pretreated biomass. The expectations regarding the organosolv lignin nanoparticles were confirmed in the Pickering emulsion tests, which presented not only a higher emulsification index but also high stability, even in the presence of salts. The success in directing the microdevice for preparing nanoparticles sparked interest in performing the Pickering emulsion in an integrated and in situ manner. In summary, the present study confirms the need to explore low-molecular-weight PEG in organosolv pretreatments for the fractionation of biomass.

## Supplementary Material



## Data Availability

Data and materials are available on request.

## Notes

Figure S1- Images of crude organosolv lignin (left) and crude acid lignin (right); Figure S2- Optical microscope images of toluene/water, soybean oil/water, n-octanol/water systems stabilized by organosolv lignin nanoparticles; Figure S3- Cumulative droplet size distribution of different Pickering systems; Figure S4- Images of Pickering emulsion systems with different solvents; and Figure S5- Cumulative droplet size distribution of n-octanol/water systems stabilized by lignin nanoparticles (PDF)
