# Supplementary material for: Production of Glucose from PEG-Based Pretreated Green Coconut Fiber and Synthesis of Lignin Nanoparticles for Stabilization of O/W Pickering Emulsion
Source: ACS Omega. 2025 Nov 14;10(46):55806–15. doi: 10.1021/acsomega.5c07310 (PMC12658658; doi:10.1021/acsomega.5c07310)

Production of glucose from PEG-based pretreated green coconut fiber and synthesis of lignin nanoparticles for stabilization of o/w Pickering emulsion

Cleitiane da Costa Nogueira[1]; Amanda Gabriela Viana Fabrício[1]; Gabriela Guimarães Lourenço[1]; Íthalo Barbosa Silva de Abreu[2]; Thelma Sley Pacheco Cellet[3]; Emmanuel Damilano Dutra[2]; Jackson Araújo de Oliveira[1]; Domingos Fabiano de Santana Souza[1]; Carlos Eduardo de Araújo Padilha[1]*

[1]Laboratory of Alternative Energy and Transport Phenomena, Chemical Engineering Department, Federal University of Rio Grande do Norte (UFRN), Natal-RN, Brazil.

[2]Research Group On Biomass Energy, Department of Nuclear Energy, Federal University of Pernambuco (UFPE), Recife-PE, Brazil.

[3]Polymeric and Composite Materials Group, Department of Chemistry, State University of Maringá (UEM), Maringá-PR, Brazil.

*email: domingos.fabiano@ufrn.br

SUPPORTING INFORMATION

Figure S1. Images of crude organosolv lignin (left) and crude acid lignin (right).

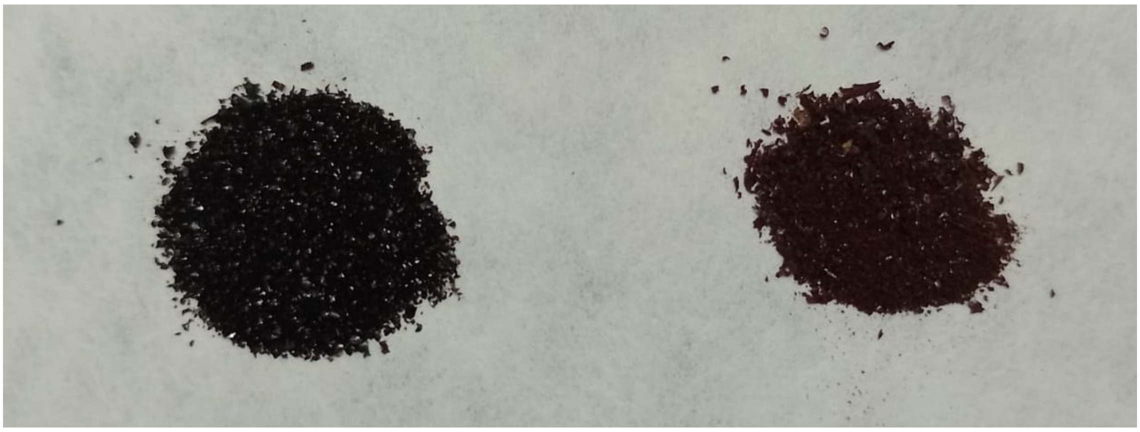

Figure S2. Optical microscope images of toluene/water (1 g/L -A, 2 g/L -B, 5 g/L LNP-C), soybean oil/water (1 g/L -D, 2 g/L -E, 5 g/L LNP-F), n-octanol/water (1 g/L -G, 2 g/L -H, 5 g/L LNP-I) systems stabilized by organosolv lignin nanoparticles.

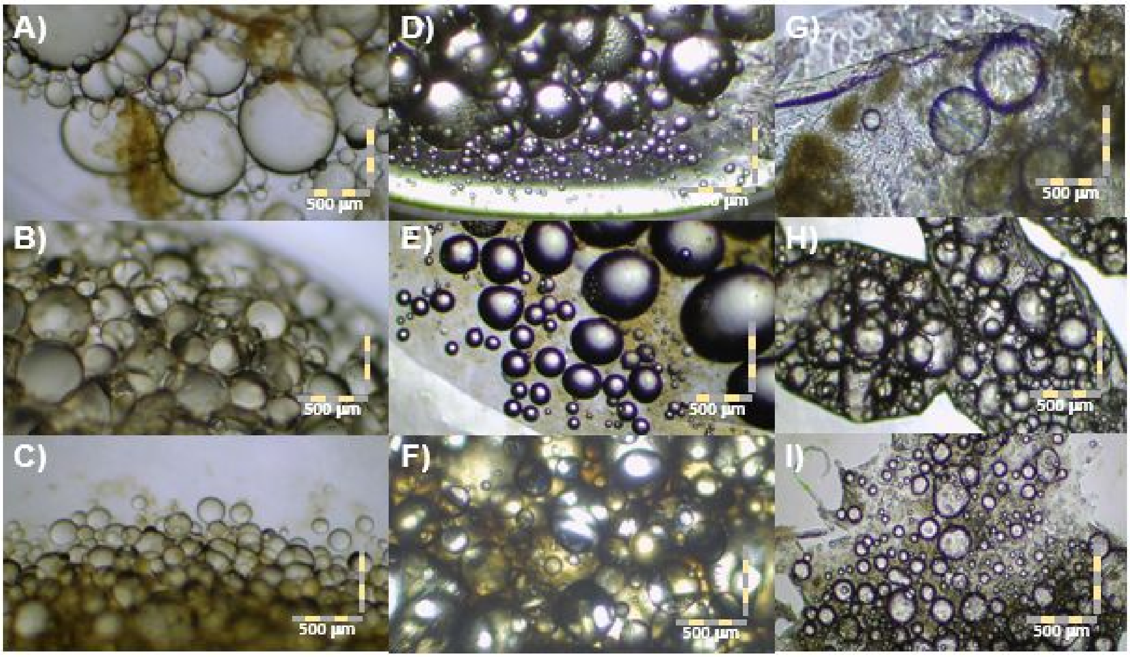

Figure S3. Cumulative droplet size distribution of toluene/water (A), soybean oil/water (B), and n-octanol/water (C) systems stabilized by different dosages of organosolv lignin LNPs.

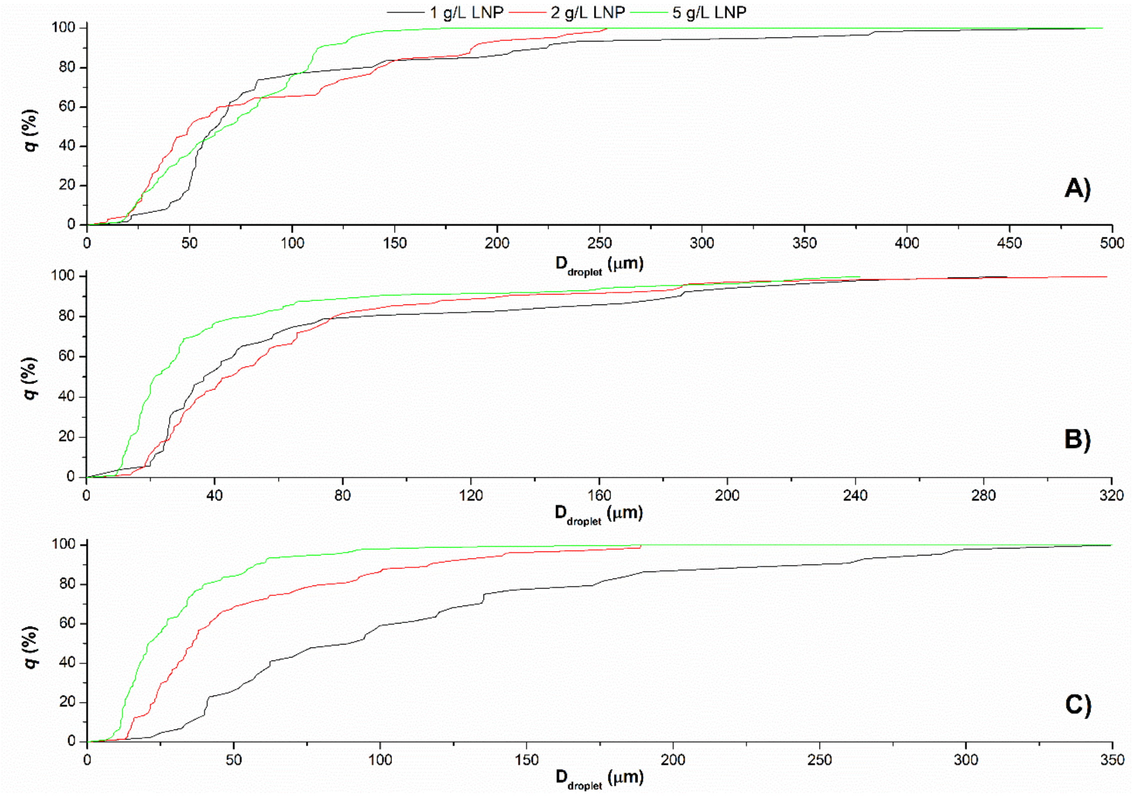

Figure S4. Images of Pickering emulsion systems with toluene (A for organosolv lignin; D for acid lignin), soybean oil (B for organosolv lignin; E for acid lignin), and n-octanol (C for organosolv lignin; F for acid lignin)after 14 days of preparation. The LNP concentrations used were 0.5, 1.0, 2.0, and 5.0 g/L (from left to right) in each type of Pickering emulsion system.

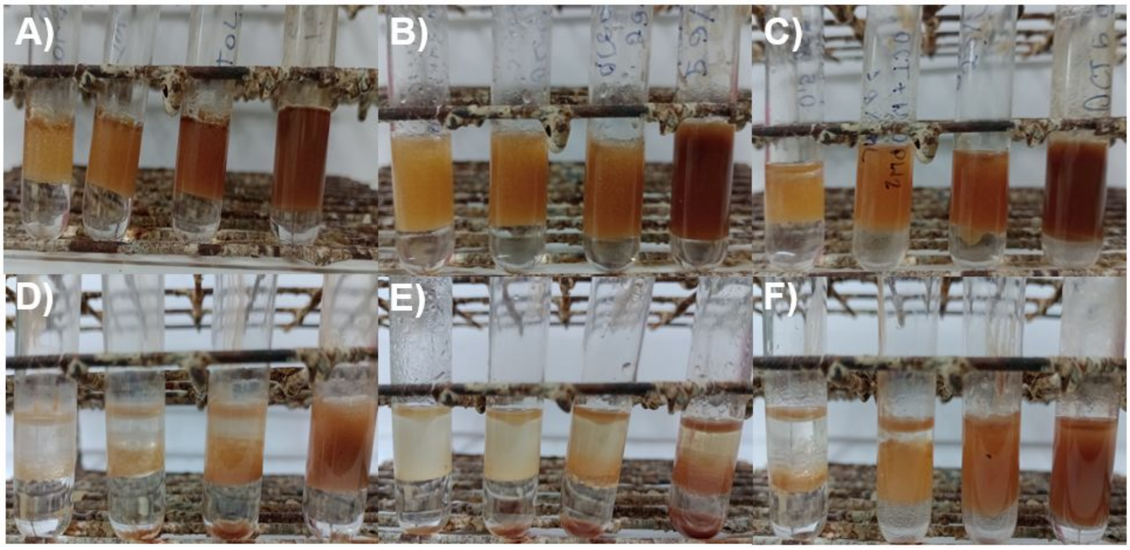

Figure S5. Cumulative droplet size distribution of n-octanol/water systems stabilized by organosolv lignin nanoparticles (A) and acidic lignin nanoparticles (B) in the presence of sodium chloride.

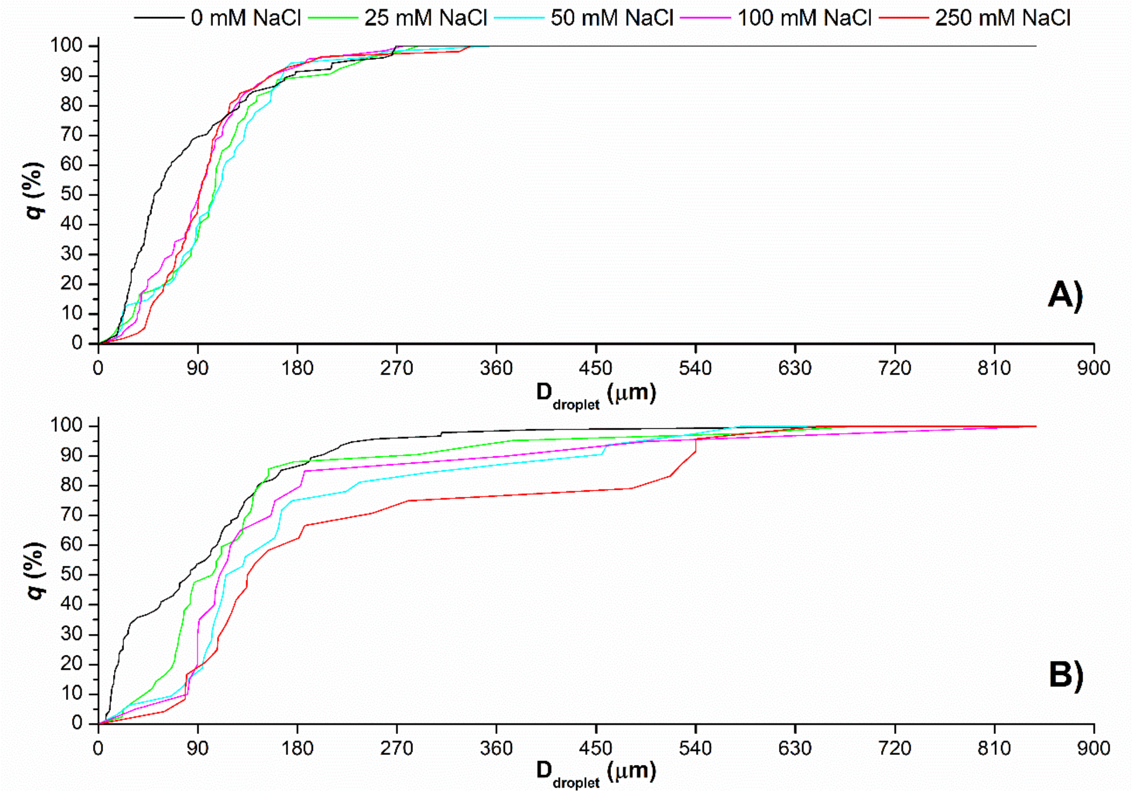

Supplement: Supplementary file 1 [file ao5c07310_si_001.pdf]
